# Boston Medical and Surgical Journal

**Published:** 1844-09

**Authors:** 


### BIBLIOGRAPHICAL NOTICES.

*Boston Medical and Surgical Journal.*—The editor of this well known and deservedly popular Journal, in the No. for August 7th, announces the commencement of the *thirty-first* volume. We congratulate the editor upon the continued success of his enterprise. It would indeed be strange if after this long experience and with the high tone and spirit which he infuses into his pages, any rival could detract from his well-earned reputation. We subjoin a few of his remarks:

"Ours is now the only one in the United States which is published weekly, having survived, unharmed, the rivalry of no less than three publications of the same class. It requires something more than a prospectus or operations, to maintain a medical journal. There is necessarily a fearful outlay of capital, quite discouraging at first; and when there is taken into account the great number of losses annually occurring, very few, it is presumed, would be willing to enter anew upon the business, after having had experience in permanently establishing one. Unlike other periodicals, its subscribers are of necessity only here and there one out of hundreds and thousands, and then they are spread so widely over the entire face of the Union, that collections are always difficult. Still, under all the aspects of the case, we have passed on till the commencement of this *thirty-first volume.* We hope for the continued good will, and the literary and scientific assistance of our brethren. With their countenance, and our own continued exertions, the Journal will pursue its quiet way, without ostentation, or a presumptuous display unbecoming the legitimate object to which it is expressly devoted, or the character it has attained."

